# Genetic risk of type 2 diabetes modifies the effects of a lifestyle intervention aimed at the prevention of gestational and postpartum diabetes

**DOI:** 10.1007/s00125-022-05712-7

**Published:** 2022-04-30

**Authors:** Emilia Huvinen, Jari Lahti, Miira M. Klemetti, Paula H. Bergman, Katri Räikkönen, Marju Orho-Melander, Hannele Laivuori, Saila B. Koivusalo

**Affiliations:** 1 Teratology Information Service, Department of Emergency Medicine and Services, Helsinki University Hospital and University of Helsinki, Helsinki, Finland; 2 Department of Psychology and Logopedics, University of Helsinki, Helsinki, Finland; 3 Department of Obstetrics and Gynecology, Helsinki University Hospital and University of Helsinki, Helsinki, Finland; 4 Biostatistics Consulting, Department of Public Health, University of Helsinki and Helsinki University Hospital, Helsinki, Finland; 5 Department of Clinical Sciences in Malmö, Lund University, Malmö, Sweden; 6 Department of Obstetrics and Gynecology, Tampere University Hospital, Tampere, Finland; 7 Center for Child, Adolescent, and Maternal Health Research, Faculty of Medicine and Health Technology, Tampere University, Tampere, Finland; 8 Institute for Molecular Medicine Finland, Helsinki Institute of Life Science, University of Helsinki, Helsinki, Finland; 9 Medical and Clinical Genetics, University of Helsinki and Helsinki University Hospital, Helsinki, Finland; 10 Department of Obstetrics and Gynecology, Turku University Hospital and University of Turku, Turku, Finland

**Keywords:** Diet, Gene–environment interaction, Genetic risk, Gestational diabetes, Lifestyle intervention, Physical activity, Polygenic risk score, Prevention, Type 2 diabetes

## Abstract

**Aims/hypothesis:**

The aim of this study was to assess the interaction between genetic risk and lifestyle intervention on the occurrence of gestational diabetes mellitus (GDM) and postpartum diabetes.

**Methods:**

The RADIEL study is an RCT aimed at prevention of GDM and postpartum diabetes through lifestyle intervention. Participants with a BMI ≥30 kg/m^2^ and/or prior GDM were allocated to intervention and control groups before pregnancy or in early pregnancy. The study visits took place every 3 months before pregnancy, once in each trimester, and at 6 weeks and 6 and 12 months postpartum. We calculated a polygenic risk score (PRS) based on 50 risk variants for type 2 diabetes.

**Results:**

Altogether, 516 participants provided genetic and GDM data. The PRS was associated with higher glycaemic levels (fasting glucose and/or HbA_1c_) and a lower insulin secretion index in the second and third trimesters and at 12 months postpartum, as well as with a higher occurrence of GDM and glycaemic abnormalities at 12 months postpartum (*n* = 356). There was an interaction between the PRS and lifestyle intervention (*p*=0.016 during pregnancy and *p*=0.024 postpartum) when analysing participants who did not have GDM at the first study visit during pregnancy (*n* = 386). When analysing women in tertiles according to the PRS, the intervention was effective in reducing the age-adjusted occurrence of GDM only among those with the highest genetic risk (OR 0.37; 95% CI 0.17, 0.82). The risk of glycaemic abnormalities at 12 months postpartum was reduced in the same group after adjusting additionally for BMI, parity, smoking and education (OR 0.35; 95% CI 0.13, 0.97).

**Conclusions/interpretation:**

Genetic predisposition to diabetes modifies the response to a lifestyle intervention aimed at prevention of GDM and postpartum diabetes. This suggests that lifestyle intervention may benefit from being tailored according to genetic risk.

**Clinical trial registration:**

ClinicalTrials.gov identifier: NCT01698385

**Graphical abstract:**

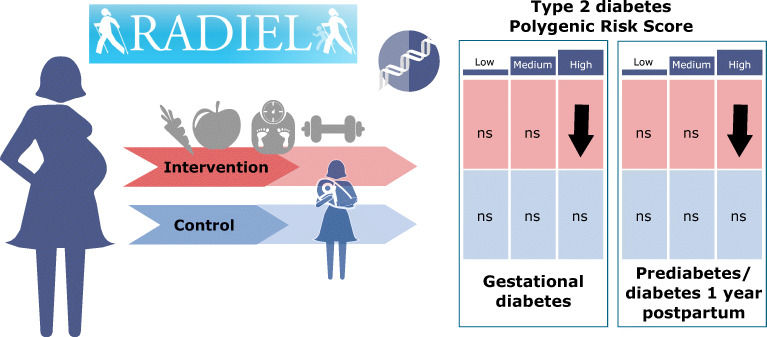

**Supplementary Information:**

The online version contains peer-reviewed but unedited supplementary material available at 10.1007/s00125-022-05712-7.

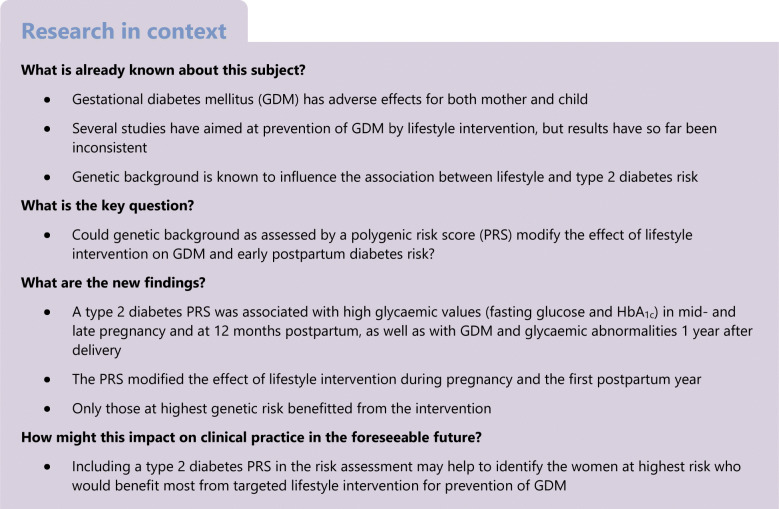



## Introduction

Gestational diabetes mellitus (GDM) is the most common pregnancy complication, with prevalence rates ranging from 1 to 28% [1], with adverse effects for the mothers and children involved. It results in increased short- and long-term morbidity, from perinatal complications such as macrosomia, birth trauma and increased Caesarean section rates [2], to elevated risk of diabetes and adiposity later in life, in both the mother and the child [3]. As women with prior GDM are at a tenfold higher risk of developing type 2 diabetes [4], there is an urgent need for prevention.

Lifestyle intervention has been successful in slowing, or even preventing, progression to type 2 diabetes in high risk individuals [5], and specifically among women with prior GDM [6]. However, the results of GDM prevention studies have been less consistent [7]. Some obtained positive results [8, 9] whereas others failed in their attempt [10]. In the RADIEL study (the Finnish Gestational Diabetes Prevention Study), lifestyle intervention succeeded in preventing GDM only in specific subgroups [8], and not among those recruited before pregnancy, for example [11]. The most recent Cochrane review [7] stated that the current evidence for a possible benefit from lifestyle intervention is at most modest, and that varying diagnostic criteria and heterogeneous study populations challenge the conclusions. The authors called for further research to disentangle the specific effects of lifestyle intervention in diverse groups of women.

One possible contributor to the discordant results may be genetic susceptibility. Studies over recent decades have provided interesting insights into the interplay between our genome and lifestyle. With regard to type 2 diabetes, several studies have revealed gene–environment interactions between specific SNPs and certain dietary factors and physical activity (PA), as well as weight reduction intervention [12]. Similarly, in the RADIEL study, we demonstrated how the *MTNR1B* polymorphism affected individual responses to lifestyle intervention [13]. Lately, the focus has shifted to the use of polygenic risk scores (PRS) [14], which enable calculation of the genetic risk based on multiple SNPs. However, it is not known whether the genetic risk of type 2 diabetes, as assessed by a PRS, affects the individual response to pregnancy lifestyle intervention aimed at prevention of GDM and early postpartum diabetes.

As lifestyle intervention requires both financial and human resources, it is crucial to identify those people benefitting the most from preventive efforts. Therefore, the aim of this study was to explore to what extent a type 2 diabetes PRS is associated with the glycaemic health of women at high risk of GDM, and whether the genetic risk modifies the effect of lifestyle intervention aimed at prevention of GDM and postpartum diabetes.

## Methods

### Study design and participants

This study is a secondary analysis of the randomised controlled intervention study RADIEL, which was performed during the years 2008–2013 in Helsinki University Hospital in Helsinki, Finland, and South Karelia Central Hospital, Lappeenranta, Finland. Previous publications have presented the details of the study [8, 15, 16].

The study participants were 18 years of age or older, and had a BMI ≥30 kg/m^2^ and/or a history of GDM. Multiple pregnancy, communication problems (based on language skills, for example), physical disabilities, medications or diagnoses affecting glucose metabolism, severe psychiatric diagnoses and current substance abuse led to exclusion. Women were recruited either before or in early pregnancy before 20 weeks of gestation, and allocated to an active lifestyle intervention group or a control group.

The study complied with the Declaration of Helsinki. All participants entered the study voluntarily, signed an informed consent form and were free to discontinue the study at any stage. The study was approved by the ethics committees of Helsinki University Hospital (14 September 2006, Dnro 300/E9/06) and South Karelia Central Hospital (11 September 2008, Dnro M06/08), and it was registered at ClinicalTrials.gov (clinical trial registration number NCT01698385).

### Intervention

The combined lifestyle intervention, provided by trained study nurses, aimed at prevention of GDM among women at high diabetes risk. The study visits took place every 3 months before pregnancy, once in each trimester, and at 6 weeks and 6 and 12 months postpartum. The intervention group received individualised advice on increasing moderate-intensity PA to 150 min/week and limiting gestational weight gain. The dietary advice followed the Nordic dietary recommendations [17], and focused mainly on increasing the intake of fibre, vegetables, fruits and berries, as well as decreasing the intake of saturated fats. The control group attended the study visits for measurements but received only standard care such as general leaflets usually provided by antenatal clinics.

### Measurements

At each study visit, anthropometric measurements were obtained and venous blood samples were taken. The biochemical analyses at each visit included assessments of glucose metabolism (HbA_1c_, fasting glucose, fasting insulin) and lipid metabolism (cholesterol, LDL-cholesterol, HDL-cholesterol, triacylglycerols), and inflammatory markers (high-sensitivity C-reactive protein). A 2 h 75 g OGTT was performed at enrolment, in the first and second trimester of pregnancy (unless GDM was diagnosed earlier), and at 6 weeks and 12 months postpartum. Participants with prior bariatric surgery or those with known diabetes diagnosis did not receive an OGTT. Background questionnaires covered socioeconomic status, lifestyle (e.g. smoking and medications), previous pregnancies, and family history of diabetes and cardiovascular diseases. Smoking in any trimester of pregnancy was recorded as ‘smoking during pregnancy’.

PA was self-reported as minutes of at least moderate-intensity PA per week. Based on the data from food frequency questionnaires, we calculated a healthy food intake index describing the quality of the diet overall. The maximum score was 18, with points for each nutritional goal: intake of high-energy/low-nutrient snacks (0, 1 or 2 points), sugar-sweetened beverages (0 or 1 points), fast food (0 or 1 points), high-fibre grains (0, 1 or 2 points), fat spread (0, 1 or 2 points), low-fat cheese (0 or 1 points), low-fat milk (0, 1 or 2 points), fish (0, 1 or 2 points), red and processed meat (0, 1 or 2 points), vegetables (0, 1 or 2 points), and fruits and berries (0 or 1 points). A higher score indicated a healthier diet.

### Genotyping and calculating the type 2 diabetes polygenic risk score

DNA was extracted from whole blood samples from 537 participants using a Maxiprep kit (Qiagen, Valencia, CA, USA). We genotyped 336 SNPs associated with type 2 diabetes, obesity or hyperlipidaemia using a Sequenom iPLEX platform (Sequenom, San Diego, CA, USA) in the year 2014.

We used PLINK 1.9 software (http://pngu.mgh.harvard.edu/~purcell/plink/) for genotype quality control and clumping [18]. We used the following parameters for clumping of the genotype data: *p* value threshold 1, linkage disequilibrium threshold (*r*^2^) 0.5, clumping window width 250 kb. Prior to clumping, we excluded all SNPs with a minor allele frequency <0.05, genotyping rate <0.9 and Hardy–Weinberg equilibrium *p* value <1 × 10^−4^. We also excluded samples if data on >10% of SNPs were missing. After quality control, there were 537 samples with genotype data on 195 SNPs. We used PRSice 2.1 [19] to calculate the PRS, using the genotype quality control settings recommended by the software developers [20]. For the SNP weights, we used the effect-size estimates obtained from Xue et al [21]*.* We applied a *p* value threshold of 5 × 10^−8^ for including type 2 diabetes-associated SNPs in the PRS. This resulted in inclusion of 50 SNPs in the PRS.

### Outcomes

The Finnish Current Care Guidelines provided the thresholds for diagnosing GDM based on 2 h 75g OGTT: 0 h ≥ 5.3 mmol/l, 1 h ≥10.0 mmol/l and 2 h ≥8.6 mmol/l [22]. One value exceeding any of the cut-offs led to a GDM diagnosis, and exceeding these thresholds in the first trimester led to a diagnosis of early GDM. ‘Booking GDM’ refers to a GDM diagnosis at enrolment (mean 13 gestational weeks) for the women recruited in early pregnancy.

Abnormal glucose metabolism 12 months postpartum refers to a diagnosis of either impaired fasting glucose (fasting glucose 6.1–6.9 mmol/l), impaired glucose tolerance (2 h glucose 7.8–11.0 mmol/l) or type 2 diabetes (fasting glucose ≥7.0 mmol/l or 2 h glucose ≥11.1 mmol/l). Alternatively, prior physician-diagnosed diabetes led to registration of a glycaemic abnormality. The indices for insulin resistance (HOMA-IR) and insulin secretion (HOMA-B) used the equations according to Matthews et al [23].

We also calculated a success score based on the example in the Finnish Diabetes Prevention Study [5], and modified the components of the score on achievement of the predefined lifestyle goals specific to this study. The maximum score was 5, consisting of the following: increasing fibre intake to 30 g or more (0 or 1 points), consumption of five or more portions of fruits, berries and vegetables per day (0 or 1 points), intake of saturated fats less than 10% of daily energy intake (0 or 1 points), gestational weight gain adequate or less than adequate according to the US Institute of Medicine’s guidelines [24] (0 or 1 points), and self-reported duration of moderate-intensity PA per week of 150 min or more (0 or 1 points). The definition of a successful intervention was three or more points.

### Statistical analyses

The data are presented as mean values with SD, medians with IQR, or as frequencies with percentages. We used the Shapiro–Wilk test to examine the normal distribution of the variables. The χ^2^ test, Fisher’s exact test, Mann–Whitney *U* test, Kruskal–Wallis test, ANOVA, or independent-samples *t* test were used for between-group comparisons as appropriate.

Associations between the PRS and glycaemic markers were assessed using linear regression, and logistic regression was used when analysing the association with glycaemic diagnoses. Additionally, we included an interaction term in the regression analyses to detect the possible effect of an interaction between a type 2 diabetes PRS and lifestyle intervention on GDM incidence or the incidence of glycaemic abnormalities 12 months postpartum. In the case of any significant PRS × intervention interaction effects, we also assessed the SNP-level interactions. These analyses were adjusted for age.

To compare the effects of the intervention according to genetic risk, we divided the participants into tertiles based on their type 2 diabetes PRS: low risk (*n* = 176), medium risk (*n* = 176) and high risk (*n* = 177). The adjusted means for the occurrence of GDM and glycaemic abnormalities at 12 months postpartum were calculated using ANCOVA. All statistical tests were two-tailed.

All analyses were performed using the SPSS 24.0 software program (IBM SPSS, Chicago, IL, USA), and we considered a *p* value ≤0.05 as statistically significant.

## Results

Of the 720 originally recruited women, DNA and data on GDM diagnosis during pregnancy were available from 516 women, of whom 356 attended the study visit at 12 months postpartum. Previous publications have provided detailed flow charts of the study [11, 25]. The participants who did not provide samples for the genetic studies were similar in terms of their baseline characteristics such as pre-pregnancy BMI, age, parity, occurrence of GDM and early GDM, PA and diet at baseline, as well as GDM history. However, they were more likely to smoke during pregnancy (10.3% vs 4.6%, *p*=0.020).

Among the participants included in this substudy, 130 presented with ‘booking GDM’, i.e. were already diagnosed at enrolment. There were no statistically significant differences between the intervention and control groups in terms of their age, pre-pregnancy BMI, socioeconomic status and parity. Additionally, there were no differences in the occurrence of GDM (48.9% in the intervention group vs 48.0% in the control group, *p*=0.852), GDM treated with medication (14.9% vs 12.0%, *p*=0.329), incidence of pre-eclampsia (5.3% vs 3.5%, *p*=0.299), smoking during pregnancy (4.3% vs 6.0%, *p*=0.396), Caesarean section rate (22.5% vs 24.3%, *p*=0.627) or newborn birthweight SD (0.23 SD vs 0.38 SD, *p*=0.110). However, women in the intervention group achieved the lifestyle intervention goals (3/5) more often (18.6% vs 10.6%, *p*=0.016).

The type 2 diabetes PRS was associated with higher fasting glucose and/or HbA_1c_ throughout pregnancy and during the first postpartum year (Table 1). With respect to insulin indices, an association was evident only for HOMA-B and not for HOMA-IR. There was also an association between the PRS and GDM, early GDM and glycaemic abnormalities at 12 months postpartum (Table 2). Electronic supplementary material (ESM) Table 1 lists all the included SNPs and their individual associations with the main outcomes (GDM and glycaemic abnormalities 12 months postpartum).

**Table 1:** Age-adjusted associations between a type 2 diabetes PRS and markers of glucose metabolism during pregnancy and the first postpartum year

Time point	Markers of glucose metabolism	*N*	*p* value	*B* (95% CI)
1st trimester	HbA_1c_	430	0.100	0.02 (−0.00, 0.05)
	fP-glucose	494	0.080	0.03 (−0.00, 0.07)
	HOMA-IR	470	0.885	0.01 (−0.12, 0.14)
	HOMA-B	470	0.683	−1.50 (−8.37, 5.38)
2nd trimester	fP-glucose	502	0.005	0.05 (0.02, 0.08)
	HOMA-IR	495	0.286	−0.06 (−0.16, 0.05)
	HOMA-B	495	0.001	−10.52 (−16.61, −4.43)
3rd trimester	HbA_1c_	469	0.031	0.03 (0.00, 0.06)
	fP-glucose	483	0.027	0.04 (0.01, 0.08)
	HOMA-IR	476	0.300	−0.07 (−0.20, 0.06)
	HOMA-B	476	0.011	−13.28 (−23.56, −3.01)
6 weeks postpartum	fP-glucose	395	0.121	0.03 (−0.01, 0.07)
	HOMA-IR	361	0.945	0.00 (−0.10, 0.10)
	HOMA-B	361	0.202	−3.06 (−7.78, 1.65)
6 months postpartum	fP-glucose	398	0.036	0.04 (0.00, 0.08)
	HOMA-IR	385	0.277	−0.07 (−0.21, 0.06)
	HOMA-B	385	0.007	−7.21 (−12.42, −2.00)
12 months postpartum	HbA_1c_	350	0.034	0.03 (0.00, 0.07)
	fP-glucose	350	0.317	0.02 (−0.02, 0.07)
	HOMA-IR	332	0.757	−0.02 (−0.17, 0.12)
	HOMA-B	332	0.177	−3.51 (−8.61, 1.59)

fP-glucose, fasting plasma glucose

**Table 2:** Age-adjusted associations between a type 2 diabetes PRS and diagnoses of abnormal glucose metabolism during pregnancy and the first postpartum year

Diagnosis	*N*	*p* value	OR (95% CI)
GDM in RADIEL study	516	0.003	1.32 (1.10, 1.58)
GDM treated with medication	516	0.364	1.13 (0.87, 1.46)
Early GDM	521	0.023	1.24 (1.03, 1.50)
GDM in RADIEL study or before	520	<0.001	1.50 (1.25, 1.81)
Glycaemic abnormalities at 6 weeks postpartum	410	0.129	1.33 (0.92, 1.93)
Glycaemic abnormalities at 12 months postpartum	356	0.039	1.37 (1.02, 1.85)

**Fig. 1:**
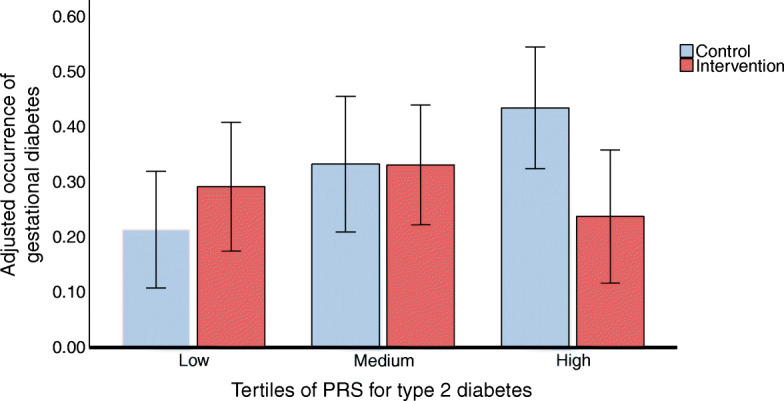
Occurrence of GDM among women in the control and intervention groups according to their genetic risk of type 2 diabetes (tertiles of PRS), adjusted for age, smoking, years of education, parity and pre-pregnancy BMI. Error bars indicate 95% CI

To assess the influence of a type 2 diabetes PRS on the effect of lifestyle intervention during pregnancy on GDM incidence, we excluded the participants with booking GDM, leaving a study population of 386 women. There was a statistically significant interaction between the PRS and the RADIEL lifestyle intervention on age-adjusted GDM incidence (*p* value for interaction = 0.014). To assess this effect further, we categorised the participants into tertiles according to their type 2 diabetes PRS: low, medium or high. Table 3 shows the characteristics of these three groups. Lifestyle intervention was effective in reducing the incidence of GDM only among women with a high type 2 diabetes PRS when adjusted for age (intervention effect OR 0.37; 95% CI 0.17, 0.82; *p*=0.014). The association remained significant after adjusting for age, pre-pregnancy BMI, parity, smoking, and years of education (intervention effect OR 0.39; 95% CI 0.17, 0.91; *p*=0.029) (Fig. 1).

**Table 3:** Characteristics of the participants in the various tertiles according to their genetic risk (PRS) for type 2 diabetes

Variable	*N*	Low-risk PRS	Medium-risk PRS	High-risk PRS	*p* value
GDM in RADIEL study	516	66 (38.8)	87 (50.0)	97 (56.4)	0.004
GDM treated with medication	516	17 (9.7)	27 (15.3)	26 (14.7)	0.253
GDM at booking	521	34 (19.8)	47 (26.7)	49 (28.3)	0.149
Early GDM	521	48 (27.9)	61 (34.7)	67 (38.7)	0.100
Abnormal glucose metabolism at 12 months postpartum	356	16 (13.8)	14 (11.4)	25 (21.4)	0.085
Pre-pregnancy obesity (BMI ≥ 30 kg/m^2^)	529	128 (72.7)	119 (67.6)	111 (62.7)	0.132
Smoking during pregnancy	469	8 (5.3)	9 (5.7)	7 (4.4)	0.856
Smoking 12 months postpartum	354	12 (10.4)	9 (7.4)	4 (3.4)	0.112
Intervention group	529	84 (47.7)	96 (54.5)	87 (49.2)	0.402
Pre-eclampsia	521	6 (3.5)	10 (5.7)	7 (4.0)	0.584
Caesarean section	521	41 (23.8)	46 (26.1)	35 (20.2)	0.423
Age in 1st trimester, years	521	31.8 ± 4.35	32.2 ± 4.84	32.5 ± 4.48	0.418
Pre-pregnancy BMI, kg/m^2^	529	32.0 ± 6.14	31.6 ± 6.11	30.6 ± 5.53	0.072
Educational attainment, years	528	14.5 ± 2.1	14.3 ± 2.0	14.7 ± 2.1	0.255
Pre-pregnancy weight, kg	529	88.1 ± 18.6	87.0 ± 18.5	85.5 ± 16.6	0.384
Weight at last visit 12 months postpartum, kg	366	88.6 ± 19.1	86.9 ± 19.1	83.5 ± 17.1	0.101
GWG, kg	392	8.99 ± 5.34	7.87 ± 6.38	8.33 ± 5.98	0.293
Fasting glucose 1st trimester, mmol/l	494	5.04 ± 0.38	5.07 ± 0.39	5.14 ± 0.42	0.059
Fasting glucose 12 months postpartum, mmol/l	350	5.37 ± 0.43	5.46 ± 0.42	5.47 ± 0.48	0.158
HbA_1c_ 1st trimester, %	430	5.25 ± 0.30	5.28 ± 0.28	5.30 ± 0.31	0.272
HbA_1c_ 1st trimester, mmol/mol	430	34 ± 3.4	34 ± 3.2	34 ± 3.6	
HbA_1c_ 12 months postpartum, %	350	5.39 ± 0.27	5.44 ± 0.30	5.49 ± 0.33	0.047
HbA_1c_ 12 months postpartum, mmol/mol	350	35 ± 3.1	36 ± 3.3	36 ± 3.7	
Fasting insulin 1st trimester, pmol/l	486	60.8 (38.4, 78.7)	51.0 (36.0, 74.3)	49.2 (32.6, 70.5)	0.072
Fasting insulin 12 months postpartum, pmol/l	336	48.6 (35.1, 70.8)	50.7 (33.3, 78.1)	47.2 (29.9, 63.5)	0.355
HOMA-IR 1st trimester	470	1.92 (1.25, 2.53)	1.66 (1.14, 2.40)	1.62 (1.02, 2.35)	0.153
HOMA-IR 12 months postpartum	332	1.71 (1.18, 2.50)	1.79 (1.09, 2.91)	1.67 (1.02, 2.28)	0.435
HOMA-B 1st trimester	470	103.4 (75.0, 148.3)	97.0 (67.4, 136.3)	86.7 (62.0, 131.0)	0.017
HOMA-B 12 months postpartum	332	85.4 (56.7, 112.4)	71.4 (53.3, 104.5)	68.6 (47.4, 97.3)	0.117
hsCRP 1st trimester, mmol/l	513	5.67 (3.55, 12.18)	5.36 (2.99, 13.06)	4.40 (2.64, 8.76)	0.012
hsCRP 12 months postpartum, mmol/l	347	1.74 (0.70, 4.00)	1.77 (0.69, 4.05)	1.39 (0.59, 2.81)	0.142
Lifestyle intervention					
HFII 1st trimester (points)	487	9.86 ± 3.09	10.17 ± 2.89	10.33 ± 2.72	0.346
HFII 12 months postpartum (points)	329	9.68 ± 3.00	10.04 ± 2.72	10.22 ± 3.07	0.388
PA 1st trimester (min/week)	446	60 (30, 135)	60 (30, 120)	80 (30, 150)	0.329
PA 12 months postpartum (min/week)	339	95 (45, 180)	90 (60, 180)	90 (40, 150)	0.637
Success score (0–5 points)	474	1 (1, 2)	1 (1, 2)	1 (1, 2)	0.463
Successful intervention (meeting 3/5 lifestyle goals)	474	23 (15.3)	25 (15.8)	20 (12.9)	0.738
Saturated fat goal		32 (24.2)	24 (17.5)	31 (24.0)	0.316
Fibre intake goal		51 (38.6)	68 (49.6)	59 (45.7)	0.186
PA > 150 min/week goal		22 (15.1)	28 (18.4)	20 (13.0)	0.416
GWG goal		89 (66.9)	94 (67.6)	82 (68.3)	0.972
Fruit, vegetables and berries (five portions)		19 (12.3)	23 (14.1)	21 (13.1)	0.887

Values are presented as *n* (%), mean ± SD or median (IQR). Percentages have been calculated using the number of participants in each tertile. For some variables, data wasn’t available for all participants

GWG, gestational weight gain; HFII, healthy food intake index; hsCRP, high-sensitivity C-reactive protein

**Fig. 2:**
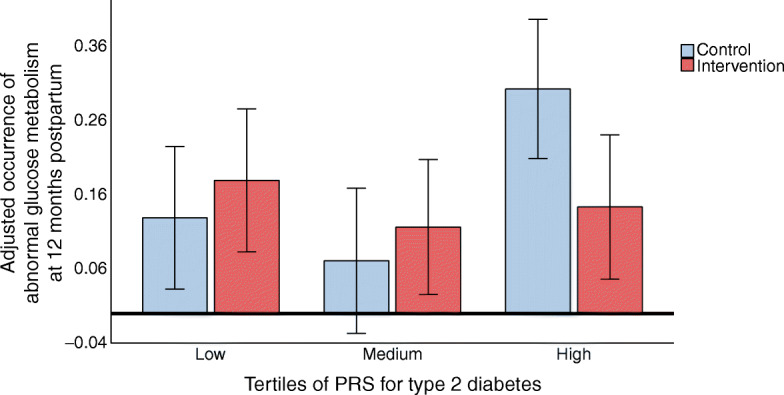
Occurrence of glycaemic abnormalities (impaired fasting glucose, impaired glucose tolerance or type 2 diabetes) at 12 months postpartum among women in the control and intervention groups according to their genetic risk of type 2 diabetes (tertiles of PRS), adjusted for age, smoking, years of education, parity and pre-pregnancy BMI. Error bars indicate 95% CI

There was also a statistically significant interaction between the type 2 diabetes PRS and the lifestyle intervention on the incidence of glycaemic abnormalities 12 months postpartum (*p* value for interaction = 0.023). When the analysis was performed using PRS tertiles, the intervention effect among women with a high type 2 diabetes PRS did not reach statistical significance when adjusted only for age (OR 0.41; 95% CI 0.16, 1.05; *p*=0.064). However, in the fully adjusted model (age, pre-pregnancy BMI, smoking and parity), the intervention was significantly associated with a reduced incidence of glycaemic abnormalities only among women with a high type 2 diabetes PRS (OR 0.35, 95% CI 0.13, 0.97, *p*=0.043) (Fig. 2). While there were a few significant individual SNP-level interactions, none of them were strong enough to drive the PRS interaction results (ESM Table 1).

## Discussion

Women with the highest type 2 diabetes PRS were most likely to benefit from lifestyle intervention for prevention of GDM in a randomised trial during pregnancy and the first postpartum year. A high PRS was associated with GDM diagnosis and markers of abnormal glucose metabolism in mid- and late pregnancy as well as 12 months postpartum. There was also an association with a lower insulin secretion index at various time points. The combined lifestyle intervention prevented GDM and postpartum glycaemic abnormalities 1 year after delivery only among the women with the highest type 2 diabetes PRS.

The RADIEL lifestyle intervention was not effective in preventing GDM or postpartum diabetes when analysing the entire study population. Many previous studies have also struggled to find an intervention effect [7]. Hence, researchers have attempted to identify discriminating factors that may explain heterogeneous responses between individuals. For example, O’Brien et al found that diet and/or lifestyle intervention had no clear effect on GDM incidence among women with a normal BMI [26]. In the meta-analysis by Madhuvrata et al, there was a reduction in GDM after diet intervention in women with risk factors for GDM [27]. Interestingly in the RADIEL study, despite having no clear impact on the primary study outcome (GDM incidence), the intervention improved the lifestyle of the participants significantly when measured using a success score. This may have potential implications. GDM is a complex metabolic disorder affecting more than just glycaemic variables, and lifestyle changes may influence the overall metabolic milieu of the fetus. In our previous study, the success score was associated with a more beneficial early growth profile of the offspring [28].

Previous studies on type 2 diabetes have suggested that genetic background may moderate individual responses to lifestyle intervention. A systematic review in 2019 reported that 28 of 66 eligible publications discovered significant gene–environment interactions [12]. These concerned specific SNPs and their interactions with either PA, diet or weight reduction interventions. For example, in the Diabetes Prevention Program and Diabetes Prevention Study, participants with the risk genotype TT in the *TCF7L2* rs12255372 showed lower type 2 diabetes incidence in the intervention group than in the control group [29, 30]. In the Diabetes Prevention Program, the lifestyle intervention was effective in reducing type 2 diabetes incidence in individuals with higher genetic risk, such as risk variants in *PPARG* [31] and *TNF* [32]. The Nurses’ Health Study II showed that, when measuring genetic risk using a PRS, the quality of the diet [33] had the strongest effect among the participants with the highest type 2 diabetes PRS. Also, among women with prior GDM, lifestyle intervention has been associated with better postpartum glycaemic levels among carriers of a risk variant in *CDKAL1* [34].

Less is known about the potential of genetic profiles to modify individual responses to lifestyle intervention aimed at GDM prevention. To our knowledge, this is the first study to report that genetic risk of type 2 diabetes modified the effectiveness of GDM lifestyle intervention. One of the few prior studies showed that women carrying the T allele of *TCF7L2* rs7903146 showed a lower incidence of GDM when adhering to a Mediterranean diet [35]. The genetic background (rs10830963 in *MTNR1B* and rs1799884 in *GCK*) also modified the association between sausage intake and GDM risk [36]. In the RADIEL study, we have previously demonstrated an interaction between the *MTNR1B* risk allele and lifestyle intervention on GDM incidence [13], but that study included only the 269 participants recruited in early pregnancy with normal glucose tolerance in the first trimester.

There are also studies that did not detect any modifying effect of distinct genetic risk profiles. In the Diabetes Prevention Program study comparing 280 women with prior GDM with 1100 control women, the PRS was positively associated with history of GDM but did not modulate the response to a lifestyle intervention aimed at prevention of type 2 diabetes [37]. However, in that study, the PRS was calculated using only 34 diabetes-associated SNPs. Many studies assessing the effect of lifestyle intervention on overall cardiovascular risk have also not seen any interaction with the PRS used [38].

Choosing a PRS based on type 2 diabetes-related SNPs appears justified, as GDM shares a partly similar genetic background. A recent meta-analysis has supported this, finding 16 variants in eight loci common to both conditions [39]. Similar to previous studies [40], the type 2 diabetes PRS [21] was associated in our study with indices suggesting deficient insulin secretion but not with insulin resistance. It was also associated with most of the glycaemic markers assessed, especially in the second and third trimesters. In fact, the performance of the PRS was remarkably good considering that we were only able to include 50 SNPs instead of the original 143 significant SNPs listed by Xue et al [21].

Many earlier PRS studies have also aimed to identify women at high risk of GDM. Several studies [37, 41–43] discovered an association between a PRS based on type 2 diabetes-related variants and glycaemic traits during pregnancy. For example, the PRS used by Kawai et al comprised 34 SNPs previously associated with type 2 diabetes [43]. These scores also identify the women with prior GDM who are at higher risk of type 2 diabetes postpartum [37, 42]. One of the largest studies assessing postpartum diabetes risk was no exception: using 59 variants and analysing a total of 2434 women of European descent with a history of GDM from two independent cohorts, it found that a higher PRS was associated with an increased risk of postpartum type 2 diabetes [33].

Importantly, we did not identify any differences between the genetic risk groups in terms of their background characteristics such as years of education and age, or lifestyle factors such as smoking, PA or healthy food intake index. This improves the reliability and interpretation of our findings. There was a lower high-sensitivity C-reactive protein (*p*=0.012) in the high-risk group, and a lower pre-pregnancy BMI (*p*=0.072), although this did not reach statistical significance. Our recruitment criterion of only including normal-weight women if they had a history of GDM offers a natural and plausible explanation for this.

Among the strengths of our study is the study design, comprising an early pregnancy randomised lifestyle intervention, with a pre-pregnancy intervention arm and continuation of the intervention until the end of the first postpartum year. The diabetes risk of GDM women is suspected to be highest during the first 5 years after pregnancy, and therefore this early postpartum period may be particularly important for the future health of these women [4]. Moreover, it is advantageous that an OGTT was performed at the start of the study, enabling us to identify those with ‘booking GDM’. This is crucial when assessing the effects of the intervention. An additional strength is the use of a PRS to assess the genetic risk instead of only specific SNPs, as it gives a more comprehensive estimate of an individual’s genetic predisposition.

One of the limitations of our study is the moderate and focused sample of analysed SNPs. Of the original 143 loci reported by Xue et al [21], only 50 were genotyped in our focused analysis based on the known variants at that time. Naturally, genome-wide data may have given a greater perspective, and most probably would have improved the accuracy of the PRS. On the other hand, our PRS was highly correlated with glycaemic values (fasting glucose and HbA_1c_) during pregnancy. It is remarkable that genotyping only 50 SNPs could be used to target lifestyle intervention for GDM and postpartum diabetes prevention. This may be an advantage in the context of limited resources. Another limitation of our study is relying solely on calculated indices when referring to insulin secretion. This is important due to the hepatic extraction of insulin prior to its appearance in the systemic circulation. As obesity alters the fraction of hepatic insulin extraction, this may affect our results. The fact that our study comprised a genetically homogeneous population of women of European descent reduces the generalisability of our findings, but this may have contributed to the fact that our results are relatively unambiguous despite our small sample size compared with most genetic studies, and we believe that the randomised intervention design and the well-documented glycaemic values and clinical variables during pregnancy and the first postpartum year counteract this limitation.

Given the constantly increasing numbers of individuals with GDM and type 2 diabetes, finding a powerful means of individualised prevention is of great importance. In the present study, those at highest genetic risk of GDM and type 2 diabetes did not differ in their background characteristics or lifestyle, demonstrating the difficulty of stratifying women based only on their clinical characteristics. Therefore, including a type 2 diabetes PRS in the risk assessment may help to identify those women at highest risk and thus benefitting most from targeted intervention. To this end, there is a need to investigate larger and more diverse ethnic populations as well as various types of interventions. Additionally, it is important to assess whether genetic susceptibility modulates the effects of lifestyle intervention on outcomes in the offspring. As GDM is also associated with a higher risk of developing type 2 diabetes and obesity among offspring, future generations may also benefit from these essential interventions.

## Supplementary information

ESM 1 (PDF 176 kb)

## Data Availability

The present informed consents do not allow archiving of clinical or register data in open repositories. Data described in the manuscript together with the codebook and the analytic code will be made available upon reasonable request. Requests regarding data availability can be addressed to the corresponding author (EH) and will be subject to further review by the national register authority and by the ethical committees.

## Abbreviations

GDM: Gestational diabetes mellitus
PA: Physical activity
PRS: Polygenic risk score
RADIEL: The Finnish Gestational Diabetes Prevention Study
