# Supplementary material for: Genetic risk of type 2 diabetes modifies the effects of a lifestyle intervention aimed at the prevention of gestational and postpartum diabetes
Source: Diabetologia. 2022 Apr 30;65(8):1291–301. doi: 10.1007/s00125-022-05712-7 (PMC9283155; doi:10.1007/s00125-022-05712-7)
Supplement: Supplementary file 1 — (PDF 176 kb) [file 125_2022_5712_MOESM1_ESM.pdf]

ESM Table 1 The age-adjusted associations of the individual SNPs in the type 2 diabetes PRS with gestational diabetes and glycaemic abnormalities 12 months postpartum in the RADIEL sample.

			Gestational diabetes				Glycaemic abnormalities 12 months postpartum			
Gene	SNP	A1	*B*	*p*	OR (95% CI)	int × SNP *p*	*B*	*p*	OR (95% CI)	int × SNP *p*
WFS1	rs10010131	A	0.08	0.544	1.09 (0.83, 1.43)	0.165	-0.19	0.400	0.82 (0.53, 1.29)	0.540
CILP2	rs10401969	C	0.16	0.544	1.18 (0.69, 2.00)	0.170	-0.43	0.368	0.65 (0.25, 1.67)	0.330
CDKNA2B	rs10811661	C	-0.27	0.178	0.77 (0.52, 1.13)	0.038	0.20	0.510	1.22 (0.68, 2.17)	0.920
KLHDC5	rs10842994	T	-0.01	0.938	0.99 (0.71, 1.38)	0.624	0.20	0.460	1.22 (0.72, 2.04)	0.630
NOTCH2	rs10923931	T	-0.05	0.796	0.95 (0.63, 1.43)	0.708	-0.22	0.542	0.80 (0.40, 1.63)	0.123
HHEX	rs1111875	T	-0.12	0.345	0.89 (0.69, 1.14)	0.052	0.12	0.568	1.13 (0.74, 1.73)	0.630
HNF1A	rs1169288	C	-0.03	0.854	0.97 (0.73, 1.30)	0.578	-0.10	0.675	0.90 (0.55, 1.47)	0.425
ADCY5	rs11708067	G	0.12	0.437	1.13 (0.83, 1.55)	0.583	-0.68	0.036	0.51 (0.27,0.96)	0.222
TCF7L2	rs12255372	T	-0.08	0.609	0.92 (0.67, 1.27)	0.097	0.20	0.428	1.22 (0.75, 2.00)	0.805
COBLL1	rs12328675	C	-0.53	0.017	0.59 (0.38,0.91)	0.110	0.27	0.400	1.31 (0.70, 2.47)	0.630
CDC123CAMK1D	rs12779790	G	-0.00	0.979	1.00 (0.74, 1.35)	0.825	0.03	0.904	1.03 (0.63, 1.68)	0.156
MC4R	rs12970134	A	0.12	0.445	1.13 (0.83, 1.54)	0.197	-0.13	0.643	0.88 (0.52, 1.50)	0.919
SLC30A8	rs13266634	T	-0.14	0.314	0.87 (0.66, 1.15)	0.267	-0.09	0.721	0.92 (0.58, 1.47)	0.806
TLE4CHCHD9	rs13292136	T	0.05	0.793	1.05 (0.72, 1.54)	0.907	0.13	0.659	1.14 (0.64, 2.03)	0.515
GRB14	rs13389219	T	-0.03	0.839	0.97 (0.74, 1.27)	0.217	-0.18	0.419	0.83 (0.53, 1.30)	0.289
CENTD2	rs1552224	C	-0.17	0.285	0.85 (0.62, 1.15)	0.174	0.12	0.612	1.13 (0.70, 1.82)	0.270
	rs2191349	G	0.00	0.973	1.00 (0.78, 1.30)	0.325	-0.11	0.598	0.89 (0.59, 1.36)	0.680
GIP	rs2291725	T	-0.17	0.179	0.84 (0.66, 1.08)	0.854	-0.44	0.039	0.65 (0.43, 0.98)	0.736
BCL11A	rs243021	A	0.17	0.192	1.18 (0.92, 1.52)	0.896	-0.12	0.590	0.89 (0.58, 1.37)	0.649
TLE1	rs2796441	A	0.02	0.889	1.02 (0.78, 1.33)	0.130	-0.03	0.913	0.98 (0.63, 1.52)	0.226
CMIP	rs2925979	T	0.03	0.821	1.03 (0.79, 1.35)	0.445	0.19	0.401	1.21 (0.78, 1.88)	0.915
IRS1	rs2972146	G	-0.19	0.472	0.91 (0.69, 1.19)	0.612	-0.48	0.056	0.62 (0.38, 1.01)	0.436
PROX1	rs340874	T	-0.29	0.027	0.75 (0.58, 0.97)	0.256	-0.22	0.292	0.80 (0.53, 1.21)	0.799
	rs4402960	T	0.30	0.028	1.34 (1.03, 1.75)	0.121	0.15	0.464	1.17 (0.77, 1.76)	0.113
ZBED3	rs4457053	G	-0.12	0.445	0.89 (0.66, 1.20)	0.642	0.32	0.181	1.37 (0.86, 2.18)	0.267
VPS13C	rs4502156	C	-0.05	0.685	0.95 (0.74, 1.22)	0.903	0.17	0.454	1.18 (0.77, 1.81)	0.902
ANKRD55	rs459193	A	-0.22	0.122	0.81 (0.61, 1.06)	0.523	-0.24	0.303	0.79 (0.50, 1.24)	0.656
GCK	rs4607517	A	0.26	0.203	1.30 (0.87, 1.94)	0.759	-0.00	0.993	1.00 (0.54, 1.84)	0.361
PABPC4	rs4660293	G	-0.05	0.739	0.95 (0.70, 1.28)	0.608	0.07	0.793	1.07 (0.65, 1.77)	0.984
KLF14	rs4731702	T	-0.12	0.379	0.89 (0.68, 1.16)	0.163	-0.30	0.210	0.74 (0.47, 1.18)	0.702
LYPLAL1	rs4846567	T	-0.19	0.174	0.83 (0.63, 1.09)	0.811	0.05	0.823	1.05 (0.67, 1.65)	0.723

ANK1	rs516946	T	0.06	0.682	1.06 (0.79, 1.44)	0.768	0.15	0.552	1.16 (0.72, 1.87)	0.230
	rs5219	T	0.10	0.445	1.10 (0.86, 1.42)	0.851	0.19	0.380	1.21 (0.80, 1.83)	0.382
ARL15	rs6450176	A	0.30	0.059	1.35 (0.99, 1.83)	0.423	0.65	0.006	1.91 (1.20, 3.03)	0.279
TMEM18	rs6548238	T	-0.55	0.007	0.58 (0.39, 0.86)	0.112	-0.59	0.118	0.55 (0.26, 1.16)	0.193
ADAMTS9	rs6795735	T	-0.11	0.401	0.90 (0.70, 1.16)	0.411	0.05	0.809	1.05 (0.69, 1.60)	0.303
GLIS3	rs7034200	A	0.12	0.324	1.13 (0.89, 1.44)	0.922	0.25	0.233	1.29 (0.85, 1.95)	0.160
HMG20A	rs7177055	G	-0.03	0.846	0.97 (0.74, 1.28)	0.529	-0.03	0.899	0.97 (0.62, 1.52)	0.602
ITPR2SSPN	rs718314	G	0.08	0.568	1.09 (0.82, 1.45)	0.358	0.14	0.580	1.14 (0.71, 1.84)	0.302
CTRB2	rs7202877	G	0.20	0.318	1.22 (0.83, 1.79)	0.317	0.56	0.049	1.75 (1.00, 3.05)	0.714
HNF1B	rs7501939	T	0.14	0.289	1.15 (0.89, 1.50)	0.350	-0.13	0.549	0.87 (0.56, 1.36)	0.183
THADA	rs7578597	C	-0.23	0.460	0.80 (0.44, 1.45)	0.909	-1.03	0.166	0.36 (0.08, 1.53)	0.998
GCKR	rs780094	T	-0.20	0.131	0.82 (0.63, 1.06)	0.928	0.08	0.715	1.08 (0.71, 1.64)	0.858
PRC1	rs8042680	A	-0.27	0.049	0.77 (0.59, 1.00)	0.301	-0.04	0.862	0.96 (0.63, 1.48)	0.252
GIPR	rs8108269	G	-0.03	0.850	0.98 (0.75, 1.27)	0.628	0.39	0.062	1.47 (0.98, 2.21)	0.305
JAZF1	rs864745	T	0.00	1.000	1.00 (0.78, 1.29)	0.020	0.00	0.990	1.00 (0.67, 1.51)	0.520
TP53INP1	rs896854	T	0.01	0.935	1.01 (0.78, 1.30)	0.793	0.23	0.273	1.26 (0.84, 1.90)	0.147
VEGFA	rs9472138	T	-0.27	0.056	0.77 (0.58, 1.01)	0.653	-0.22	0.340	0.80 (0.50, 1.27)	0.108
	rs9686661	T	-0.02	0.918	0.98 (0.66, 1.45)	0.084	-0.65	0.106	0.52 (0.24, 1.15)	0.101
TFAP2B	rs987237	G	-0.07	0.643	0.93 (0.69, 1.26)	0.346	0.21	0.374	1.24 (0.77, 1.98)	0.937

A1 = effect allele, *B* = regression coefficient, OR = OR, 95% CI = 95% CI,
int × SNP = interaction between a SNP and RADIEL lifestyle intervention
